# Serum Levels of Adropin Improve the Predictability of MELD and Child-Pugh Score in Cirrhosis: Results of Proof-of-Concept Clinical Trial

**DOI:** 10.3389/ti.2023.11176

**Published:** 2023-06-02

**Authors:** Yotam Kolben, Ariel Kenig, Asa Kessler, Yuval Ishay, Sarah Weksler-Zangen, Mualem Eisa, Yaron Ilan

**Affiliations:** ^1^ Faculty of Medicine, Hebrew University, Jerusalem, Israel; ^2^ Department of Medicine, Hadassah Medical Center, Jerusalem, Israel

**Keywords:** cirrhosis, MELD, Child-Pugh score (CPS), adropin, fibrosis

## Abstract

Adropin is a peptide that was suggested to have a role in cirrhosis. The present study aimed to determine the ability to use serum adropin levels to improve their prediction accuracy as an adjunct to the current scores. In a single-center, proof-of-concept study, serum adropin levels were determined in thirty-three cirrhotic patients. The data were analyzed in correlation with Child-Pugh and MELD-Na scores, laboratory parameters, and mortality. Adropin levels were higher among cirrhotic patients that died within 180 days (1,325.7 ng/dL vs. 870.3 ng/dL, *p* = 0.024) and inversely correlated to the time until death (*r* ^2^ = 0.74). The correlation of adropin serum levels with mortality was better than MELD or Child-Pough scores (*r* ^2^ = 0.32 and 0.38, respectively). Higher adropin levels correlated with creatinine (*r* ^2^ = 0.79. *p* < 0.01). Patients with diabetes mellitus and cardiovascular diseases had elevated adropin levels. Integrating adropin levels with the Child-Pugh and MELD scores improved their correlation with the time of death (correlation coefficient: 0.91 vs. 0.38 and 0.67 vs. 0.32). The data of this feasibility study suggest that combining serum adropin with the Child-Pugh score and MELD-Na score improves the prediction of mortality in cirrhosis and can serve as a measure for assessing kidney dysfunction in these patients

## Graphical Abstract

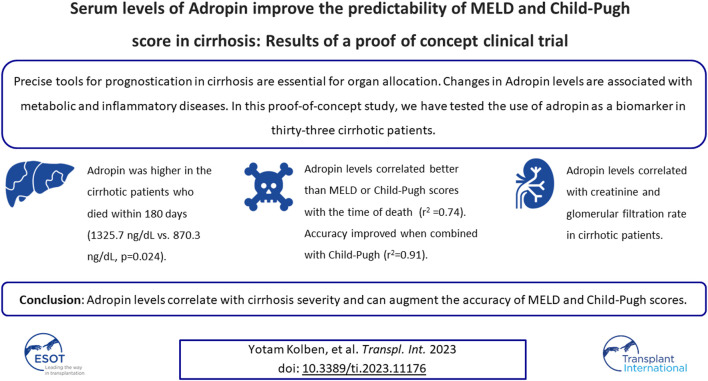

## Introduction

Cirrhosis is a progressive fibrosing nodular condition that disrupts the entire typical architecture of the liver [1]. More than 160 million people worldwide had cirrhosis in 2017, and more than 0.8 million patients with cirrhosis die yearly [2]. Liver transplantation is currently the only curative therapy available. Prioritizing liver allocation in non-acute liver failure is based on mortality prediction scores. Precise organ allocation is crucial in the face of organ shortage, which accounts for a large proportion of wait-list mortality [3]. Accurate prognostication is essential for coordinating patients’ expectations, assessing therapeutic risk-benefit balance, and more.

The Child-Pugh score (CPS) classification and the Model for end-stage liver disease (MELD) score are commonly used models for predicting mortality in cirrhosis [4]. However, these models have several drawbacks. Ascites and encephalopathy included in the CPS classification are subjective and may be variable according to the physician’s judgment and the use of diuretics and lactulose. The international normalized ratio (INR) does not sufficiently reflect coagulopathy and liver function and is variable throughout different laboratories [4]. Although adding sodium to MELD enhances its performance [5], improving the currently available methods for assessing the degree of severity and predicting prognosis in chronic liver disease remains an unmet need [6–12].

Adropin is a 76-amino-acids-secreted peptide encoded by the Enho gene and is conserved among humans, mice, and rats [13]. The physiological role of Adropin in the liver is unknown. High levels of Adropin correlated with a low incidence of type 2 diabetes mellitus (DM), elevated HDL cholesterol, lower BMI, LDL cholesterol, triglyceride levels, and blood pressure [14–16]. Preliminary data suggested that serum levels of Adropin may be related to the degree of disease severity in cirrhotic humans [17].

The present single-center proof-of-concept study aimed to determine the potential of using serum levels of adropin as a prognostic biomarker in patients with chronic liver disease and to determine its use as an adjunct to MELD and CPS for improving their performance in predicting mortality.

## Patients and Methods

### Ethical Considerations

This single-center prospective, observational study was approved by the Institutional Review Board (IRB) (Hadassah medical center 0634-19-HMO, NCT04660409). Participants signed informed consent during enrollment as defined by the local IRB.

### Study Population

Adult subjects (18–80 years) with chronic liver disease of all etiologies were enrolled. The main exclusion criteria were evidence of other acute severe disease or any acute medical condition within 48 h of blood tests. Controls were adults without known liver disease.

### Serum Adropin Concentration and Clinical Data

A single blood test for serum levels of adropin was obtained. Serum was collected using a serum separator tube and centrifuged for 20 min at 1,000 × g. Samples were stored in aliquots at −80°C. Adropin levels were determined using a sandwich enzyme immunoassay kit for adropin (Cloud Clone Corp., Katy, TX, United States) according to the manufacturer’s procedure. The optical absorbance was measured spectrophotometrically at 450 nm.

Subjects’ clinical and laboratory data were generated from the patient’s medical records.

### Statistical Analysis

Comparing adropin serum levels between cirrhotic and non-cirrhotic patients was carried out using the non-parametric Mann-Whitney test. The same test was used to compare different scoring systems trying to predict mortality. ROC analysis was used when the score was significantly different between groups. The Kruskal-Wallis non-parametric test was applied to compare Adropin levels between three independent groups. The non-parametric tests were used due to the small sample size and the non-normal distribution of adropin levels in some subgroups compared. The Pearson correlation coefficient was calculated to assess the strength of the linear association between adropin levels and other quantitative variables. All tests applied were two-tailed, and a *p*-value of 0.05 or less was considered statistically significant.

## Results

### Patient Characteristics

Clinical and laboratory data were obtained from 33 cirrhotic patients (Table 1). Sixteen (48.5%) of the patients were females, with a higher female rate among patients without nonalcoholic steatohepatitis (NASH, 64.7% vs. 31.3%). The common etiology was NASH, assigned to 16 (48.5%) patients. It was followed by hepatitis B and hepatitis C with five (15.2%) and four (12.1%) cases each. Eight patients were assigned with more than one diagnosis.

**TABLE 1:** Patients characteristics.

Characteristic	Etiology		Total (*N* = 33)
	NASH (*N* = 16)	Other (*N* = 17)	
Adropin levels–ng/dL	973.1 ± 136.1	934.3 ± 89.7	953.1 ± 79.3
Female sex–no. (%)	5 (31.3)	11 (64.7)	16 (48.5)
Age–yr	67.9 ± 2.4	67.0 ± 2.6	67.7 ± 1.8
Etiology^a^–no. (%)			
NASH	16 (100)	0 (0.0)	16 (48.5)
AIH	2 (12.5)	6 (35.3)	8 (24.2)
HBV	0 (0)	5 (29.4)	5 (15.2)
HCV	1 (6.3)	3 (17.6)	4 (12.1)
Biliary cirrhosis^b^	1 (6.3)	1 (5.9)	2 (6.1)
DILI	1 (6.3)	1 (5.9)	2 (6.1)
Other^c^	1 (6.3)	4 (23.5)	5 (15.2)
Hepatocellular Carcinoma–no. (%)	5 (31.3)	4 (23.5)	9 (27.3)
Child-Pugh group–no. (%)			
A	1 (6.3)	5 (29.4)	6 (18.2)
B	12 (75.0)	8 (47.1)	20 (60.6)
C	3 (18.8)	4 (23.5)	7 (21.2)
MELD-Na score–mean	18.1 ± 1.5	14.1 ± 1.4	16.0 ± 1.24
Sodium–mmol/L (136–145)	133.2 ± 1.0	133.8 ± 0.9	133.5 ± 0.7
Bilirubin–µmol/L (5–21)	40.7 ± 7.9	70.2 ± 38.0	55.9 ± 19.8
INR (0.9–1.2)	1.34 ± 0.05	1.26 ± 0.03	1.3 ± 0.03
Creatinine–µmol/L (62–115)	116.9 ± 17.1	103.1 ± 12.4	109.8 ± 10.4
Albumin–gr/L (32–48)	31.7 ± 1.7	31.3 ± 1.4	31.5 ± 1.1

Abbreviations: AIH, autoimmune hepatitis; DILI, drug-induced liver injury; HBV, hepatitis B virus; HCV, hepatitis C virus; INR, international normalized ratio; MELD, a model for end-stage liver disease; NASH, nonalcoholic steatohepatitis.

^a^ For eight patients, cirrhosis was attributed to more than one diagnosis.

^b^ Including one patient with primary sclerosing cholangitis and one with primary biliary cirrhosis.

^c^ Including patients with alcoholic hepatitis (who also had NASH, diagnosis), cardiac cirrhosis, sickle cell disease (one each), and two cryptogenic cirrhosis.

The average MELD-sodium (MELD-Na) score was 16.0, corresponding with most patients (60.6%) in Child-Pough group B. The severity was marginally higher in patients with NASH cirrhosis, albeit not reaching significance (MELD-Na 18.1 vs. 14.1, *p* = 0.39). Hepatocellular carcinoma (HCC) was diagnosed in 27.3%. Five-of the HCC patients had NASH, and four with viral hepatitis.

The laboratory values are described in Table 1. Patients with NASH cirrhosis had insignificantly lower bilirubin levels (40.7 µmol/L vs. 75.1 µmol/L, *p* = 0.47) and higher creatinine levels (116.9 µmol/L vs. 103.1 µmol/L, *p* = 0.51).

### Serum Adropin Levels in Patients With Chronic Liver Disease

Serum adropin levels were higher among patients with chronic liver disease relative to controls, albeit not reaching statistical significance (953.1 ng/dL vs. 735.0 ng/dL, *p* = 0.37).

Among the patients with chronic liver disease, subjects with NASH and viral-mediated liver disease had similar serum levels of adropin, 973.1 ng/dL and 946.4 ng/dL, respectively. Lower serum Adropin levels were noted among patients with autoimmune hepatitis relative to all other diagnoses (818.9 ng/dL vs. 996.0 ng/dL, *p* = 0.38). Patients with chronic liver diseases not attributed to the above causes had non-significantly higher adropin levels (1,112.6 ng/dL). It included patients diagnosed with biliary cirrhosis, DILI, cardiac cirrhosis, sickle cell disease, and cryptogenic cirrhosis. Finally, adropin levels were not affected by the development of HCC (938.2 ng/dL vs. 958.7 ng/dL, *p* = 0.54).

Adropin levels were higher in males with chronic liver disease (1,109.8 ng/dL vs. 786.6 ng/dL, *p* = 0.056), and including the healthy controls, the sex difference widened (1,067.3 ng/dL vs. 779.2 ng/dL, *p* = 0.049).

### Correlation of Serum Adropin Levels and Kidney Functions

**FIGURE 1:**
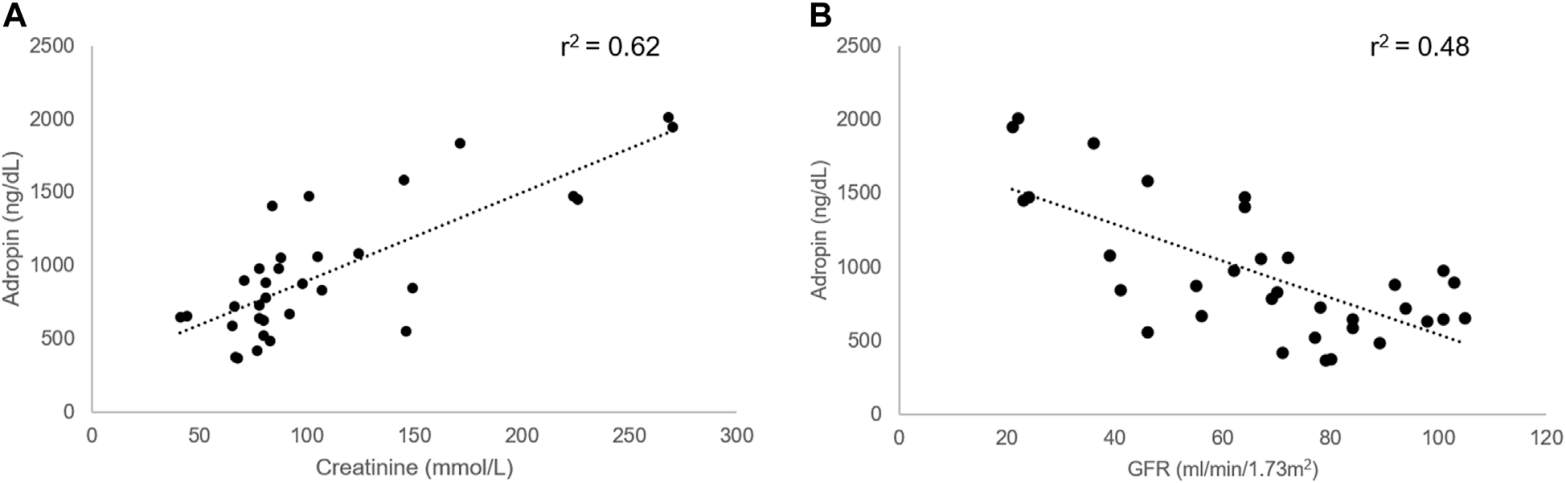
Correlation between adropin and kidney function in cirrhotic patients. A higher level of adropin correlated with concomitant higher creatinine levels **(A)** and lower GFR **(B)** in cirrhotic patients. Abbreviations: GFR, glomerular filtration rate.

An association between adropin levels and serum creatinine levels was documented. Patients with higher creatinine levels had higher adropin levels (*r* ^2^ = 0.62, p < 0.001). The glomerular filtration rate (GFR) was calculated according to the 2021 CKD-EPI equation. Calculated GFR also correlated with adropin levels (*r* ^2^ = 0.48, *p* = 0.032, Figure 1).

### Correlation of Serum Adropin Levels and AFP

A negative correlation trend was noted with alpha-fetoprotein (AFP, *r* ^2^ = −0.34, *p* = 0.2).

No correlation was found with age, bilirubin, INR, sodium levels, or albumin.

### Correlation of Serum Adropin Levels With Concomitant Disease

**FIGURE 2:**
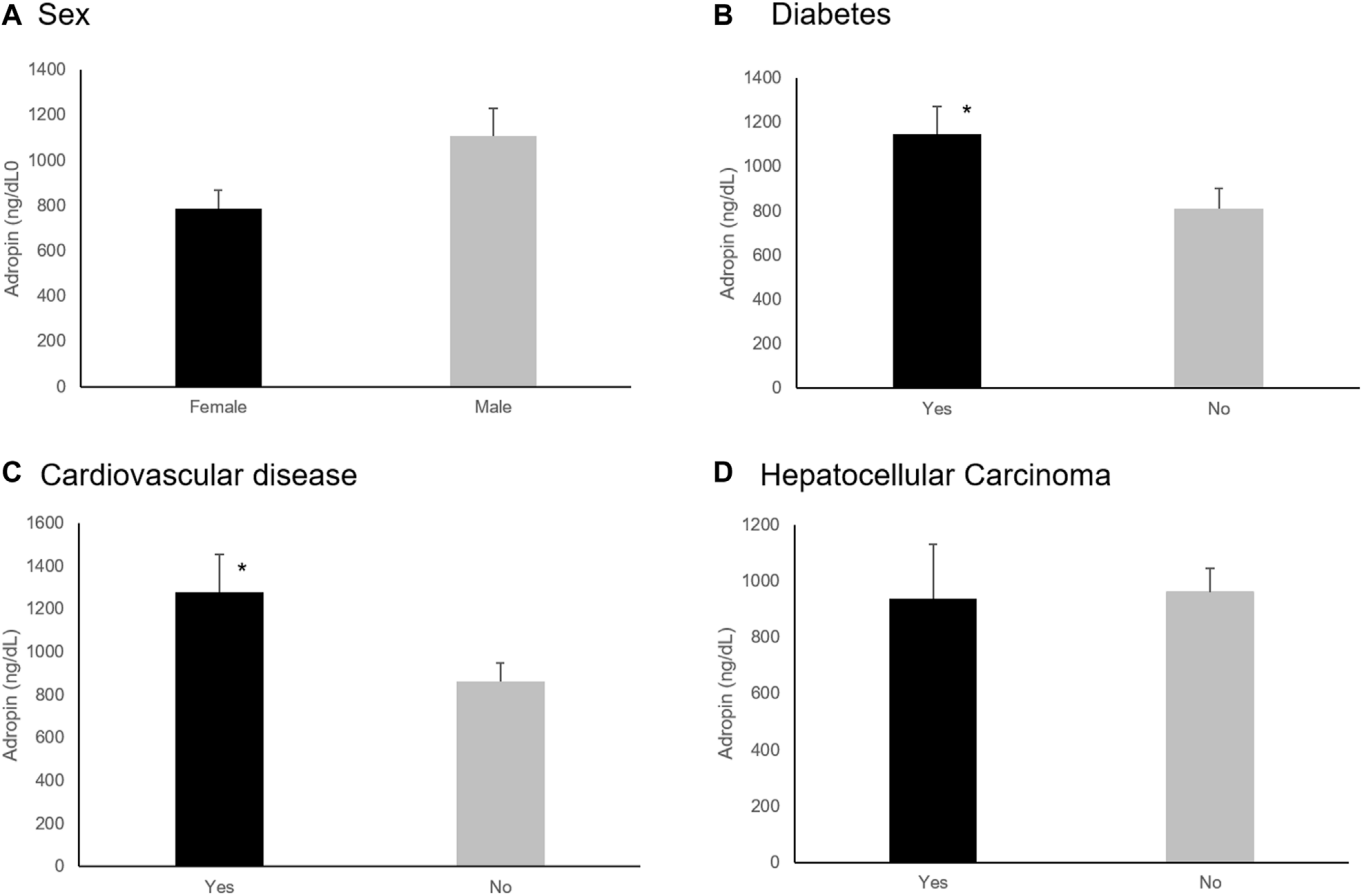
Effect of sex and underlying medical conditions on Adropin levels. **(A)** Females had lower levels of Adropin (*p* = 0.056). **(B)** Diabetes Mellitus and **(C)** cardiovascular disease diagnosis increased adropin levels significantly (*p* = 0.035 and *p* = 0.031, respectively). **(D)** the presence of Hepatocellular carcinoma did not affect adropin levels. *p < 0.05.

Underlying medical conditions impacted the adropin levels regardless of the etiology of chronic liver disease. Patients with DM had significantly higher Adropin levels (1,148.0 ng/dL vs. 810.5 ng/dL, *p* = 0.035). A similar effect was noted among patients with cardiovascular diseases (1,279.5 ng/dL vs. 865.2 ng/dL, *p* = 0.031). No effect was noted in patients with hypertension and hyperlipidemia (Figure 2).

### Serum Levels of Adropin Predict Mortality in Patients With Chronic Liver Disease and Improve the MELD-Na and CPS Predictability

**FIGURE 3:**
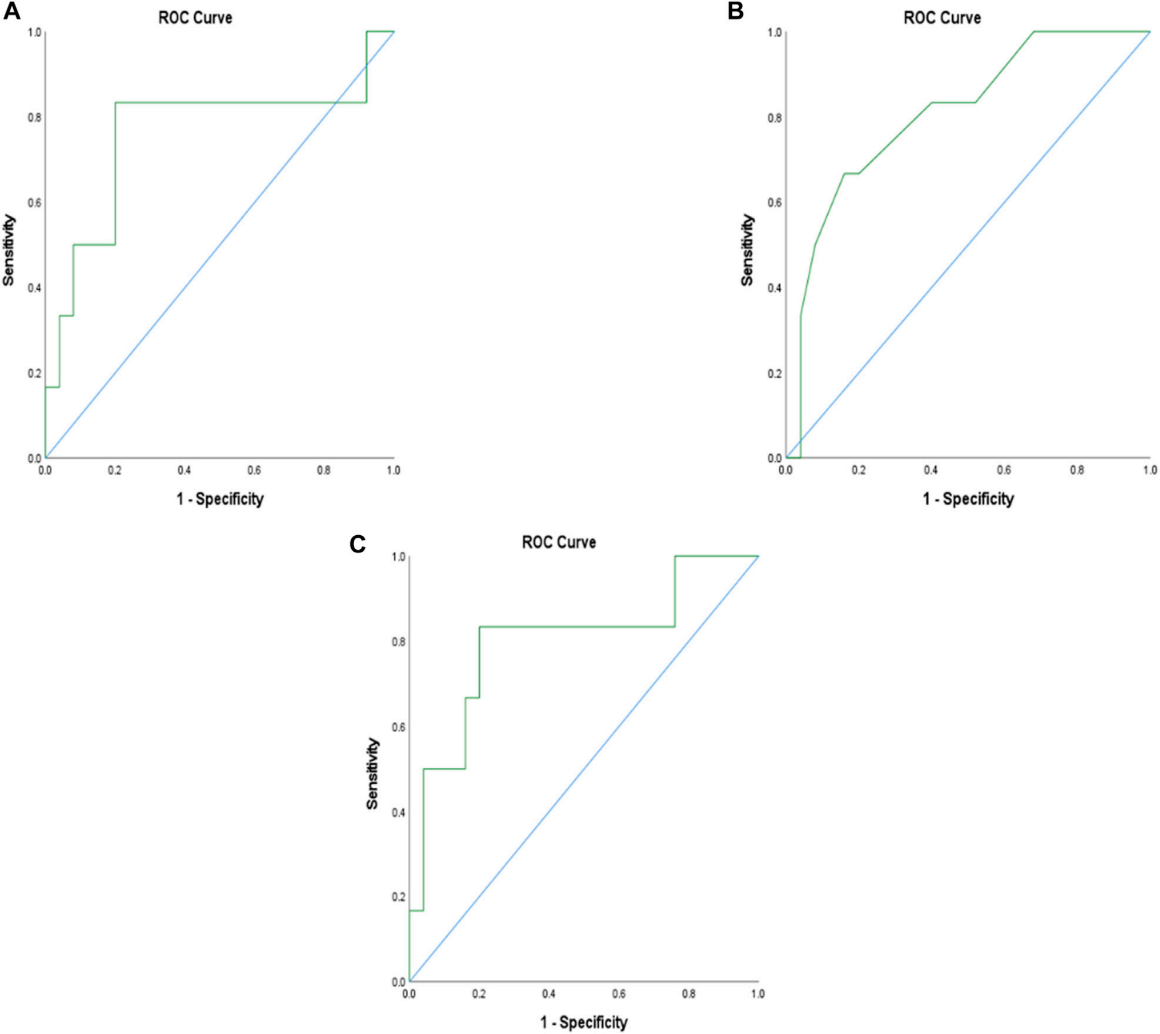
ROC analysis of adropin, MELD-Na, and adropin with Child-Pugh integration score to predict mortality within 180 days. **(A)** ROC analysis of adropin; AUC = 0.76, **(B)**, ROC analysis of MELD-Na; AUC = 0.8 **(C)** ROC analysis of the integration of adropin and Child-Pugh score; AUC = 0.80. Abbreviations: AUC, the area under the curve; MELD, a model for end-stage liver disease.

Eight patients (24.2%) died between 7 and 233 days following enrollment in the study. Adropin levels were higher among the six patients that died within 180 days (1,325.7 ng/dL vs. 870.3 ng/dL, *p* = 0.024). The area under the curve (AUC) in ROC analysis for adropin to predict mortality within 180 days was 0.76. When implementing adropin levels of 1,058 ng/dl as a cut-off point, sensitivity and specificity for death during the following 180 days were 83% and 80%, respectively. MELD-Na, but not CPS, was also significantly higher in patients who died 6 months after being tested (21.2 vs. 14.9, *p* = 0.043) with an AUC of 0.8. An integration of adropin levels with the CPS ( $\left.Child-Pugh\;score\times Adropin/1000\right)$ increased 180 days mortality differentiation, with higher values predicting mortality ( $\frac{CPS\times Adropin}{1000}$ , no units, 11.95 vs. 6.87, *p* = 0.006), with an AUC of 0.8 (Figure 3). When implementing a cut-off point of 8.54 (no units), the sensitivity and specificity of predicting death within the next 180 days were 83% and 80%, respectively, identical to adropin alone.

**FIGURE 4:**
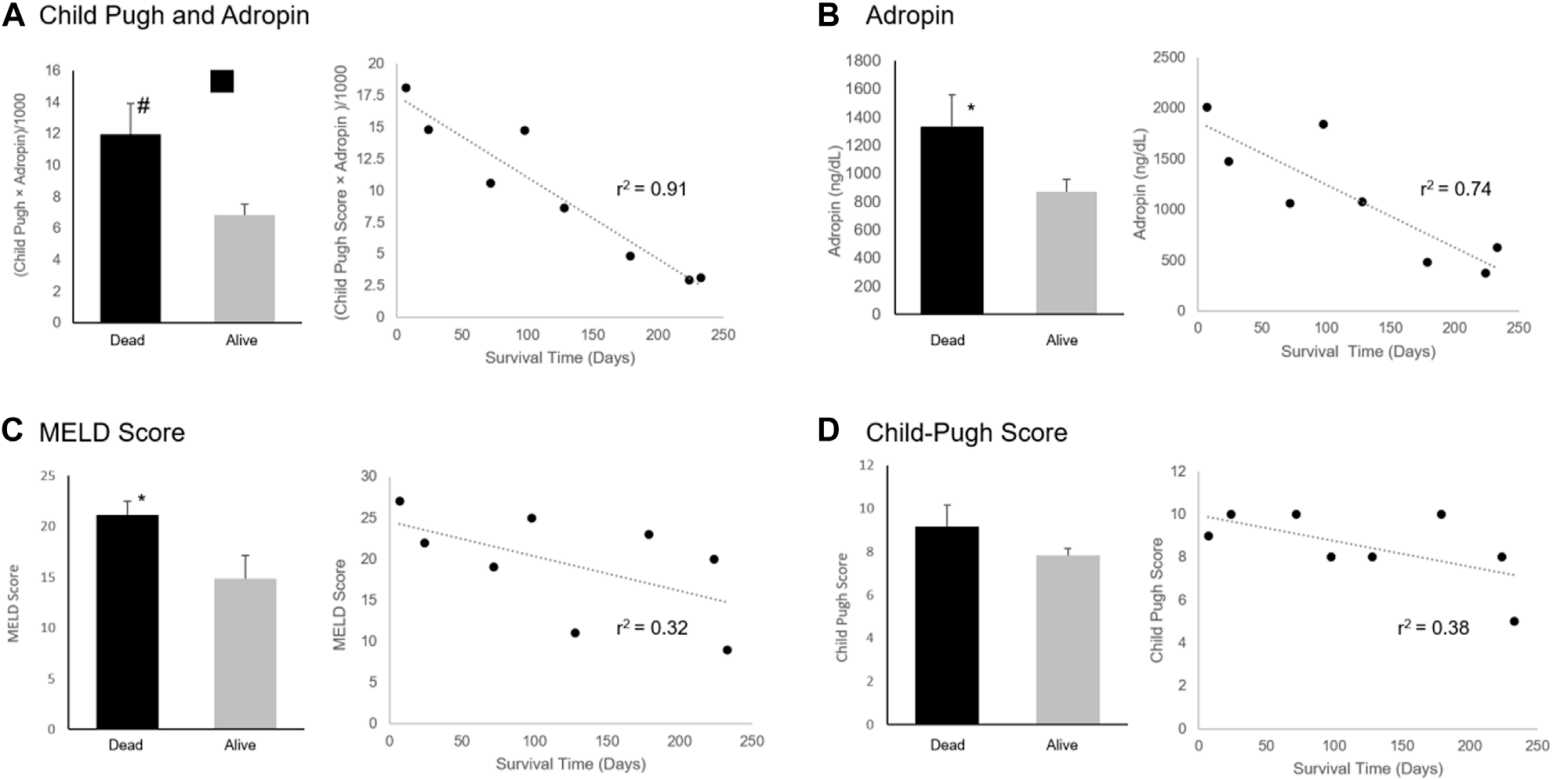
Adropin, MELD Score, and Child-Pugh Score Association with Mortality. Difference between surviving and mortality after 180 days and correlation with time of death of **(A)** An integration of adropin and Child-Pugh score, **(B)** Adropin levels, **(C)** MELD-Na score, and **(D)** Child-Pugh score. Abbreviations: MELD, a model for end-stage liver disease. #*p* < 0.01, *p < 0.05.

Adropin levels were inversely correlated to the time until death (*r* ^2^ = 0.74) (Figure 4). Adropin levels correlated better than MELD-Na or CPS with patients’ survival time (*r* ^2^ = 0.32 and 0.38, respectively). An integration of adropin levels with the CPS ( $\left.Child-Pugh\;score\times Adropin/1000\right)$ had excellent correlation with the time of death (*r* ^2^ = 0.91).

An integration of Adropin levels and MELD-Na ( $\left.MELD-Na\;score\times Adropin/1000\right)$ showed a trend for improved prediction of the 180 days mortality (23.64 in deceased vs. 7.17 in alive, *p* = 0.082). Correlation with the time of death was not as precise (*r* ^2^ = 0.67) as the combination with CPS (Figure 4).

## Discussion

This feasibility single-center trial showed that serum levels of Adropin predicted mortality better than the often-used MELD-Na and CPS. Moreover, adding Adropin levels to the Child-Pugh and MELD-Na scores improved mortality prediction. Serum levels of adropin levels positively correlated with poor prognostic factors such as mortality and kidney injury.

Serum adropin levels decreased and negatively correlated with liver injury in NASH mice. Knockout of Adropin significantly exacerbated hepatic steatosis, inflammatory responses, and fibrosis in mice. Administration of Adropin bioactive peptides slowed NASH progression in mice [18]. In humans, in a study with 99 patients with alcoholic cirrhosis, serum Adropin levels correlated positively with disease severity [17]. Higher Adropin levels were found in hepatocytes of patients with chronic hepatitis with a higher degree of fibrosis [19]. This data correlates with our findings. Interestingly, elevated Adropin levels were found in patients with systemic sclerosis, suggesting a role for Adropin in the fibrosis process, which may be different from its role in other metabolic processes [20].

Previous studies demonstrated that higher adropin levels correlated with a better metabolic profile [14, 21]—the present study associated higher levels with DM and cardiovascular diseases. The difference may be explained by the potential effect of cirrhosis on adropin levels.

The correlation between adropin and creatinine was significant. Previous studies regarding adropin and kidney function focused mainly on diabetic nephropathy and showed a negative correlation between adropin and nephropathy progression [22, 23]. Hepatorenal syndrome is a common and severe complication of cirrhosis, with limited treatment options. Much of its pathophysiology beyond circulatory dysfunction is yet to be defined [24]. The present study’s data suggest that adropin may play a role in this pathologic process.

This study is limited by the relatively small number of patients and being a single-center study. The significance of the data supports extensive studies for determining the potential role of adropin as a biomarker and therapeutic target in these patients. In addition, the exact cause of death was unknown, preventing us from inferring about adropin relation to cirrhosis-related deaths. However, as cirrhosis is a significant driver of morbidity and mortality, an association with all-cause mortality is essential for medical decision-making.

In summary, elevated serum adropin levels are a poor prognostic factor in patients with chronic liver disease independent of the etiology. Adropin was superior to the standard prognostic models, CPS and MELD-Na, in predicting mortality and correlated with decreased renal function in cirrhotic patients. It can serve as a variable for improving the prediction performance of current scores. Larger cohorts are expected to shed light on the potential use of adropin as an additional biomarker to diagnose better and predict the prognosis of chronic liver disease and as a potential new therapeutic target in cirrhosis and hepatorenal syndrome.

## Data Availability

The raw data supporting the conclusion of this article will be made available by the authors, without undue reservation.

## Abbreviations

AUC, area under the curve; CPS, Child-Pugh score; DM, diabetes mellitus; DILI, drug-induced liver injury; HCC, hepatocellular carcinoma; INR, international normalized ratio; IRB, institutional review board; MELD, model for end-stage liver disease; MELD-Na, Na-model for end-stage liver disease; NAFLD, nonalcoholic fatty liver disease; NASH, GFR nonalcoholic steatohepatitis glomerular filtration rate.
